# Adenoma characteristics associated with post-polypectomy proximal colon cancer incidence: a retrospective cohort study

**DOI:** 10.1038/s41416-022-01719-4

**Published:** 2022-02-11

**Authors:** Rhea Harewood, Kate Wooldrage, Emma C. Robbins, James Kinross, Christian von Wagner, Amanda J. Cross

**Affiliations:** 1 Department of Epidemiology and Biostatistics, School of Public Health, Imperial College London, London, UK; 2 Cancer Screening and Prevention Research Group (CSPRG), Department of Surgery and Cancer, Imperial College London, London, UK; 3 Department of Surgery and Cancer, Imperial College London, London, UK; 4 Research Department of Behavioural Science and Health, University College London, London, UK

**Keywords:** Epidemiology, Cancer epidemiology, Cancer screening, Colorectal cancer, Cancer prevention

## Abstract

**Background:**

Colorectal cancer (CRC) screening is less effective at reducing cancer incidence in the proximal colon compared to the distal colorectum. We aimed to identify adenoma characteristics associated with proximal colon cancer (PCC).

**Methods:**

Endoscopy and pathology data for patients with ≥1 adenoma detected at baseline colonoscopy were obtained from 17 UK hospitals between 2001 and 2010. Multivariable Cox regression models were used to estimate adjusted hazard ratios (aHRs) and 95% confidence intervals (CIs) for PCC, and, for comparison, distal CRC incidence, by adenoma characteristics.

**Results:**

Among 18,431 patients, 152 and 105 developed PCC and distal CRC, respectively, over a median follow-up of 9.8 years. Baseline adenoma characteristics positively associated with PCC incidence included number (≥3 vs. < 3: aHR 2.10, 95% CI: 1.42–3.09), histology (tubulovillous/villous vs. tubular: aHR 1.61, 95% CI: 1.10–2.35) and location (any proximal vs. distal only: aHR 1.70, 95% CI: 1.20–2.42), for which there was borderline evidence of heterogeneity by subsite (*p* = 0.055). Adenoma dysplasia (high vs. low grade) was associated with distal CRC (aHR 2.42, 95% CI: 1.44–4.04), but not PCC (p-heterogeneity = 0.023).

**Conclusions:**

Baseline adenoma number, histology and proximal location were independently associated with PCC and may be important to identify patients at higher risk for post-polypectomy PCC.

## Background

The incidence of colorectal cancer (CRC) is higher in individuals with a personal history of adenomas [1, 2]. Reductions in incidence have been achieved through colonoscopies to identify and remove adenomas, known precursors, followed by post-polypectomy surveillance colonoscopy to prevent the progression of missed, incompletely resected or de novo adenomas to malignancy. These methods have been particularly effective at preventing cancers in the distal colon and rectum, but unfortunately have demonstrated a weaker protective effect in the proximal colon, with a greater propensity for adenoma recurrence [3–6] and post-colonoscopy CRC [7–9] in this subsite.

One reason for the lower level of protection offered by colonoscopy against proximal colon cancer is likely to be the failure to identify those patients at higher risk for proximal colon cancer after polypectomy, who would benefit from referral to colonoscopy surveillance. In the case where such patients are identified, failure to provide surveillance at optimum intervals could be another contributing factor.

There are few studies reporting associations between adenoma characteristics and an increased incidence of long-term all-site CRC. A retrospective cohort study [10] in a UK population with adenomas detected at baseline colonoscopy reported associations with all-site CRC for adenoma histology and polyp location in low-risk (1–2 small [< 10 mm] adenomas at baseline) and intermediate-risk patients (3–4 small adenomas, or 1–2 adenomas with ≥1 large [≥10 mm] adenoma at baseline) and for adenoma dysplasia in intermediate- and high-risk patients (≥5 small adenomas, or ≥3 adenomas with ≥1 large adenoma at baseline). Similarly, a study in the US [11] reported associations between the number, size, histology and dysplasia of baseline adenomas and all-site CRC, with another analysis of three large prospective US cohorts finding somewhat similar results when compared to those without polyps [12]. A multi-centre population-based cohort study in Poland also found that among patients with adenomas detected, adenoma size (≥20 mm) and dysplasia were independent risk factors for all-site CRC [13].

Findings of associations between adenoma characteristics and CRC risk has been important for informing criteria for post-polypectomy risk stratification and surveillance guidelines [14–16]. However, considering the growing evidence of heterogeneity in the development of CRC by subsite, it is important to determine whether associations between adenoma characteristics and CRC differ by subsite to better understand the lower effectiveness of colonoscopy at reducing the incidence of cancer in the proximal colon compared to distal colon and rectal subsites [17–22]. This study aimed to identify baseline adenoma characteristics associated with incident proximal colon cancer in a large cohort of patients referred for colonoscopy and followed up for a median of ten years.

## Methods

### Data source

The All Adenomas study is a retrospective cohort study investigating long-term CRC incidence and the effectiveness of colonoscopic surveillance in preventing CRC among patients with adenomas. Detailed information on the study and its methodology are described elsewhere [23, 24]. Briefly, a cohort of ~250,000 patients was identified from routinely collected data from 17 UK hospitals, all known to have 6 years or more of electronic endoscopy and pathology data prior to the start of the study in 2006.

Endoscopy databases were searched for patients who underwent a lower gastrointestinal endoscopy prior to 31 December 2010. Pathology databases were searched using Systematized Nomenclature of Medicine (SNOMED) or, where not available, Systematized Nomenclature of Pathology (SNOP) codes and keywords were used to identify and classify lesions occurring in the colorectum. Automatic procedures were used to link endoscopy and corresponding pathology reports based on hospital number, name and date of birth. Manual inspection was performed to highlight any linkage issues and data were re-extracted when necessary [23].

A baseline visit was defined as the first examination at which an adenoma was detected (this may not have been a patient’s first endoscopy) and any following consecutive examinations, usually performed within 11 months, required in order to completely examine the colorectum and remove all detected adenomas. Subsequent colonic examinations were grouped into surveillance visits in a similar manner [23, 24].

Patients were included in analyses if they had a colonoscopy performed during which at least one adenoma was detected. Patients were excluded from analyses if they did not have a colonoscopy at baseline. We also excluded those with conditions which put them at a higher risk for future CRC and therefore not representative of the general population at risk. This included those with prevalent CRC, a history of inflammatory bowel disease or colitis, polyposis, juvenile polyps, hamartomatous polyps, Lynch syndrome, a family history of familial adenomatous polyposis or volvulus at baseline. Patients with a bowel resection at or before baseline or a record of colorectal carcinoma in situ from national sources more than three years prior to baseline, those with a missing endoscopy date (precluding determination of follow-up time) and those lost to follow-up (i.e. those who emigrated or could not be identified in external data sources and did not have a surveillance visit) were also excluded [23, 24]. Additionally, to ensure that we included only patients in whom the entire colorectum was examined, we excluded patients whose baseline colonoscopy was either incomplete (scope did not reach the caecum) or was of unknown completeness. We also excluded patients with a baseline colonoscopy examination prior to 2001 when UK endoscopic quality criteria were introduced. Due to small percentages of missingness for the main exposure variables (adenoma size 2.1%, adenoma histology 4.5%, adenoma dysplasia 2.4% and adenoma location 2.0%), patients missing data for any of the adenoma characteristics under study were excluded from analyses.

### Exposures at baseline colonoscopy

The following adenoma characteristics were examined at baseline: adenoma number (< 3, ≥3); adenoma size (diameter < 10 mm, ≥10 mm); adenoma histology (tubular, tubulovillous or villous); adenoma grade of dysplasia (low-grade dysplasia, high-grade dysplasia) and adenoma location (distal [between the anus and descending colon] or proximal [between the splenic flexure and caecum]). Categories for analysis were based on previous literature [2, 14], distributions in the data and taking consideration of past and current surveillance criteria [14, 25]. For individuals who had an adenoma seen at multiple endoscopy examinations during the baseline visit, summary values were assigned for each adenoma using previously published algorithms [23]. Patient and examination characteristics were also collected, including sex, age, year of examination, bowel preparation quality (excellent or good, satisfactory, poor), the presence of hyperplastic polyps (yes, no), length of the baseline visit (1 day, 2 days–3 months, >3–6 months, >6 months) and the centre where the examination was performed.

Baseline adenoma and examination quality characteristics were defined for each patient by assigning adenoma size as the largest diameter, histology as the highest degree of villousness, dysplasia as the highest grade and bowel preparation as the highest quality preparation reported.

### Outcome

Data on deaths and CRC diagnoses were obtained from NHS Digital up to 31 December 2017, and from National Health Service Central Register and National Services Scotland up to 31 May 2016. CRC diagnoses were also obtained from hospital data and were compared with those from external sources and duplicates removed. Methods used to resolve discrepancies between data sources were previously described [23]. Briefly, all CRC diagnoses from external sources and those pathologically confirmed by hospital data were counted as cancer, even if not reported by national sources for the latter. Suspected cancers reported at endoscopy but not confirmed by pathology or the national data sources were not counted as a case of cancer. Cancers were excluded if there was strong evidence they developed from an incompletely resected adenoma at baseline. These cancers were defined as those diagnosed in the same or adjacent segment of the colorectum as a large (≥15 mm) baseline adenoma seen at least twice in the 5 years before the cancer diagnosis; this was done in line with previous analyses in this dataset.

CRC was defined as adenocarcinoma of the colorectum. Proximal colon cancer included cancer between the caecum and splenic flexure (International Classification of Diseases for Oncology, third edition [ICD-O-3] codes C18.0–C18.5). Distal CRC included cancer between the descending colon and the anus (ICD-O-3 codes C18.6–C18.7; C19; C20; C21). Three patients were diagnosed with both proximal and distal cancer (one with both cancers diagnosed on the same date, two with proximal cancer diagnosed first and distal CRC diagnosed 7 months later in one and 15 months later in the other) and the proximal colon cancer was prioritised for analysis as proximal colon cancer was the primary focus of these analyses. Cancers, except those in the appendix or anus, with unknown morphology were assumed to be adenocarcinomas [23].

### Statistical analyses

The distribution of baseline patient and examination characteristics were compared within the population and by exposure variables.

Follow-up time began at the date of the last endoscopy examination in the baseline visit. Patients were censored at first diagnosis of CRC, diagnosis of volvulus, date of resection or anastomosis, death, date of complete follow-up from national sources or at 15 years of follow-up.

Cancer incidence rates per 100,000 person-years were calculated. Cumulative cancer incidence through 15 years was computed and illustrated using Kaplan–Meier survival curves and compared between exposure subgroups using the log-rank test. Joint Cox proportional hazard models with follow-up time as the underlying time metric were used to estimate hazards ratios (HRs) and 95% confidence intervals (CIs) for the association between each adenoma characteristic and proximal colon cancer or distal CRC incidence, which were treated as competing risks [26]. Wald tests were used to examine heterogeneity in the associations of adenoma characteristics with each outcome. Multivariable models were constructed, adjusting for potential confounders which included age, sex, year of examination, bowel preparation quality, presence of hyperplastic polyps, length of the baseline visit and the number of surveillance visits (treated as a time-varying variable). Models were also constructed to examine the effect of additionally mutually adjusting for all adenoma characteristics to assess which were independently associated with the outcome. Adjustment for the examination centre did not materially make a difference to the associations observed and therefore it was not included in the final models.

All variables were included as main-effect terms in the final models. Proportionality of the association between each adenoma characteristic and cancer incidence over time was assessed by a statistical test for an interaction of Schoenfeld residuals with time. There was no evidence that associations differed over time.

Two sensitivity analyses were performed. In one, patients diagnosed with both proximal colon cancer and distal CRC were excluded from analyses. In the other, due to a lack of consensus on definitions, colorectal subsites were reclassified with the proximal colon subsite redefined as the caecum to the hepatic flexure [ICD-O-3 codes C18.0–C18.3] and the distal colorectum redefined as the transverse colon to the anus [ICD-O-3 codes C18.4–C18.7; C19; C20; C21]).

In secondary analyses, associations between ‘high-risk’ findings and proximal colon cancer were examined. High-risk findings were defined according to the most recent 2020 UK surveillance guidelines as: ≥2 premalignant polyps [serrated polyps or adenomas] including ≥1 advanced colorectal polyp [a ≥10 mm serrated polyp, a serrated polyp with dysplasia, a ≥10 mm adenoma or an adenoma with high-grade dysplasia] or ≥5 premalignant polyps or a ≥20 mm non-pedunculated colorectal polyp [14]. These models were examined both with and without consideration of any additional adenoma characteristics found to be independently associated with proximal colon cancer in the main analyses.

All analyses were conducted using Stata ® version 13.1 [27]. All statistical tests were two-sided and *p*-values < 0.05 were considered statistically significant.

## Results

### Patient and examination characteristics

**Fig. 1:**
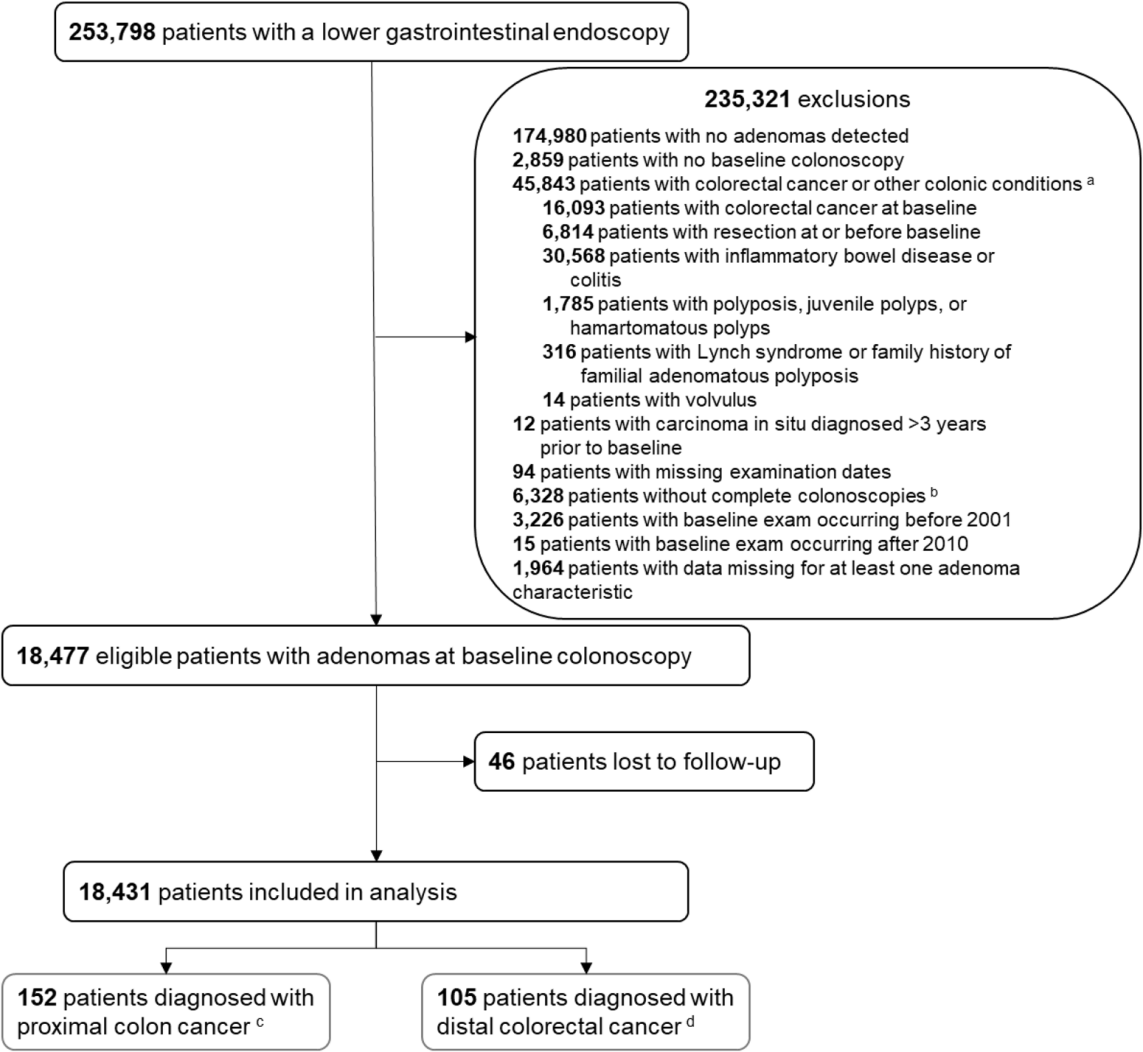
Patient flow diagram. This flow chart shows the total number of patients identified who underwent a lower gastrointestinal endoscopy along with the relevant exclusions made to arrive at the number of patients included for analysis and the number of accrued colorectal cancer cases. ^a^ conditions reported are not mutually exclusive. ^b^ a colonoscopy is considered complete if the colonoscope has reached the caecum. ^c^ proximal colon cancer incidence is defined as cancer located between the caecum and splenic flexure, International Classification of Diseases for Oncology, third edition (ICD-O-3) codes C18.0–C18.5. ^d^ distal colorectal cancer is defined as cancer located between the descending colon and the anus, ICD-O-3 codes C18.6–C18.7; C19; C20; C21.

A lower gastrointestinal endoscopy was performed in 253,798 patients. Among these, 235,321 patients were excluded, comprising 174,980 with no adenomas detected, 2859 patients with no baseline colonoscopy, 45,843 patients with CRC or other colonic conditions, 12 with carcinoma in situ diagnosed more than 3 years prior to baseline, 94 with missing examination dates, 6328 without a complete baseline colonoscopy, 3226 with a baseline examination before 2001, 15 with a baseline colonoscopy after 2010 and 1964 with missing data for at least one adenoma characteristic. Of eligible patients, 46 (0.2%) were excluded as a result of being lost to follow-up. A total of 18,431 patients remained for inclusion in analyses (Fig. 1).

Patients were followed up for a median of 9.8 years (IQR 7.4–11.9 years), during which 152 patients were diagnosed with proximal colon cancer and 105 with distal CRC; specific locations are given in Appendix Table 1.

The distribution of patient and examination characteristics in the study population and by baseline adenoma characteristics are presented in Table 1. The study population was 58.4% male, and the majority were between 55 and 74 years of age at baseline (60.0%), had their colonoscopy performed after 2005 (56.7%), had a baseline visit that spanned only 1 day (66.2%) and only 5.8% had poor bowel preparation quality (Table 1). During follow-up, over half (52.2%) of the patients had ≥1 surveillance visit (Table 1); among these patients, the time interval between visits was most commonly between >1–3 years or >3–5 years (Appendix Table 2). Patients with ≥3 adenomas, adenomas which were large (≥10 mm), tubulovillous or villous, with high-grade dysplasia or located in the proximal colon were more likely to be older or have attended their first surveillance visit within 3 years post-baseline colonoscopy compared to patients with < 3 adenomas, adenomas which were only small, tubular, with low-grade dysplasia or located distally, respectively (Table 1, Appendix Table 3). Patients with ≥3 adenomas adenomas which were large (≥10 mm), tubulovillous or villous, or with high-grade dysplasia were also more likely to have had a baseline visit performed over more than 1 day and more likely to have had follow-up surveillance compared to patients with < 3 adenomas, adenomas which were only small, tubular or with low-grade dysplasia, respectively (Table 1).

**Table 1:** Baseline patient and examination characteristics and number of follow-up visits by baseline adenoma characteristics.

			Adenoma characteristics									
			Number		Size		Histology		Dysplasia		Location	
	All patients (*N*, %)		<3	≥3	<10 mm	≥10 mm	Tubular	Tubulovillous/ villous	Low grade	High grade	Distal only	Any proximal
*Baseline characteristics*												
All patients, N (%)	18,431	100.0	15,751 (85.5)	2680 (14.5)	10,188 (55.3)	8243 (44.7)	11,562 (62.7)	6869 (37.3)	16,504 (89.5)	1927 (10.5)	10,887 (59.1)	7544 (40.9)
Sex, %												
Men	10,755	58.4	56.3	70.6	57.5	59.4	58.3	58.4	58.2	59.3	56.5	61.0
Women	7676	41.6	43.7	29.4	42.5	40.6	41.7	41.6	41.8	40.7	43.5	39.0
Age group (years), %												
<55	3586	19.5	21.1	9.8	22.5	15.7	21.7	15.7	20.3	12.2	22.7	14.8
55–64	5036	27.3	27.1	28.4	27.8	26.8	28.0	26.1	27.6	24.9	27.5	27.1
65–74	6020	32.7	31.5	39.4	30.6	35.2	31.4	34.8	32.2	36.9	31.1	35.0
≥75	3789	20.6	20.2	22.5	19.1	22.3	18.9	23.4	19.9	25.9	18.8	23.1
Year of examination, %												
2001–2005	7975	43.3	43.9	39.7	43.8	42.6	43.3	43.2	42.9	46.5	45.1	40.6
2006–2010	10,456	56.7	56.1	60.3	56.2	57.4	56.7	56.8	57.1	53.5	54.9	59.4
Bowel preparation quality, %												
Excellent or good	6490	35.2	34.6	39.0	34.0	36.7	32.7	39.4	34.9	37.5	35.1	35.3
Satisfactory	3749	20.3	20.7	18.1	20.5	20.1	19.7	21.5	20.4	20.2	21.3	18.9
Poor	1071	5.8	5.8	5.8	6.3	5.2	5.9	5.7	6.0	4.5	5.8	5.8
Unknown	7121	38.6	38.9	37.1	39.1	38.1	41.7	33.4	38.7	37.8	37.7	40.0
Length of baseline visit, %												
1 day	12,199	66.2	68.9	50.1	77.8	51.9	74.9	51.5	69.8	35.7	61.9	72.4
2 days–3 months	2800	15.2	14.7	18.4	10.1	21.4	12.1	20.4	14.4	21.8	18.4	10.6
>3–6 months	1804	9.8	8.9	15.1	6.9	13.4	7.3	14.0	8.7	19.5	10.8	8.4
≥6 months	1628	8.8	7.5	16.4	5.2	13.3	5.8	14.0	7.2	23.0	9.0	8.7
Hyperplastic polyps, %												
None/Only adenomas	14,623	79.3	80.8	70.9	78.9	79.9	78.8	80.2	79.3	80.0	80.4	77.8
≥1	3808	20.7	19.2	29.1	21.1	20.1	21.2	19.8	20.7	20.0	19.6	22.2
*Follow-up characteristics*												
Surveillance visits, %												
None	8818	47.8	49.8	36.2	53.9	40.3	51.4	41.8	49.1	37.1	47.8	47.9
1	5122	27.8	27.9	26.9	27.6	28.1	27.3	28.7	27.7	28.5	28.4	26.9
2	2980	16.2	15.3	21.2	12.5	20.7	14.3	19.2	15.6	20.8	16.4	15.8
≥3	1511	8.2	6.9	15.7	6.0	10.9	7.0	10.2	7.6	13.6	7.4	9.4

Compared to patients without a CRC diagnosis, patients with proximal colon cancer were more likely to be women, older, to have had their baseline colonoscopy prior to 2005, have a baseline visit spanning more than 1 day, to have hyperplastic polyps detected, ≥3 adenomas, a large (≥10 mm) adenoma, adenomas with tubulovillous or villous histology or proximal adenomas. They were also more likely to have fewer follow-up surveillance visits (Appendix Table 4). Compared to those with proximal colon cancer, patients with distal CRC were, however, less likely to be women, and their baseline colonoscopy was more likely to have poor bowel preparation or adenomas with high-grade dysplasia detected and less likely to have hyperplastic polyps, ≥3 adenomas or any proximal adenoma detected (Appendix Table 4).

### Baseline adenoma characteristics and proximal colon cancer or distal CRC risk

**Fig. 2:**
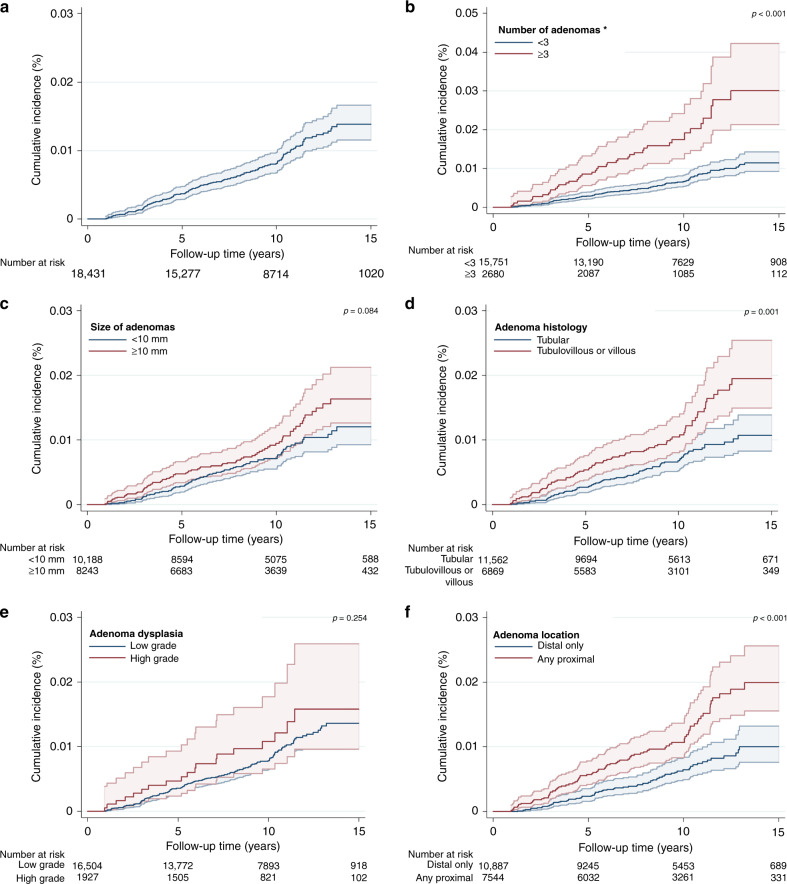
Cumulative incidence curves (%) for proximal colon cancer within 15 years of follow-up. The graphs show Kaplan-Meier survival curves for proximal colon cancer incidence: **a** overall; **b** by the number of adenomas; **c** by adenoma size (mm); **d** by adenoma histology; **e** by adenoma dysplasia; **f** by adenoma location. A different scale has been used in the graph for adenoma number and is highlighted by an asterisk. The p-values presented are for the log-rank test to compare curves for subgroups of each adenoma characteristic. The 95% CIs for each curve are shown as shaded bands.

The incidence rate of proximal colon cancer was 90 (95% CI: 77–105) per 100,000 person-years (Table 2) and overall cumulative incidence at 15 years was 1.4% (95% CI: 1.2–1.7%) (Table 2, Fig. 2). Cumulative incidence was significantly higher for patients with ≥3 adenomas compared to <3 (*p* < 0.001), adenomas with tubulovillous or villous histology compared to only tubular (*p* = 0.001) and those with any proximal compared to only distal adenomas (*p* < 0.001) (Table 2, Fig. 2).

**Table 2:** Colorectal cancer incidence by baseline adenoma characteristics.

Baseline characteristics	Patients	Cases	Person time (years)	Incidence rate per 100,000 person-years (95% CI)	Cumulative incidence (%)^a^	*p*-value^b^
*Proximal colon cancer incidence*						
All patients	18,431	152	169,143	90 (77–105)	1.4 (1.2–1.7)	
Number of adenomas						
<3	15,751	107	146,385	73 (60–88)	1.2 (0.9–1.4)	
≥3	2680	45	22,758	198 (148–265)	3.0 (2.1–4.2)	<0.001
Adenoma size (mm)						
<10	10,188	76	95,691	79 (63–99)	1.2 (0.9–1.6)	
≥10	8243	76	73,452	103 (83–130)	1.6 (1.3–2.1)	0.084
Adenoma histology						
Tubular	11,562	77	107,615	72 (57–89)	1.1 (0.8–1.4)	
Tubulovillous or villous	6869	75	61,528	122 (97–153)	2.0 (1.5–2.5)	0.001
Adenoma dysplasia						
Low grade	16,504	133	152,448	87 (74–103)	1.4 (1.1–1.7)	
High grade	1927	19	16,695	114 (73–178)	1.6 (1.0–2.6)	0.254
Adenoma location						
Distal only	10,887	67	102,967	65 (51–83)	1.0 (0.8–1.3)	
Any proximal	7544	85	66,176	128 (104–159)	2.0 (1.6–2.6)	<0.001
*Distal colorectal cancer incidence*						
All patients	18,431	105	169,143	62 (51–75)	1.0 (0.8–1.3)	
Number of adenomas						
<3	15,751	85	146,385	58 (47–72)	0.9 (0.7–1.1)	
≥3	2680	20	22,758	88 (57–136)	1.9 (0.9–4.0)	0.084
Adenoma size (mm)						
<10	10,188	52	95,691	54 (41–71)	0.8 (0.6–1.2)	
≥10	8243	53	73,452	72 (55–94)	1.2 (0.8–1.8)	0.137
Adenoma histology						
Tubular	11,562	58	107,615	54 (42–70)	0.7 (0.5–1.0)	
Tubulovillous or villous	6869	47	61,528	76 (57–102)	1.5 (1.0–2.3)	0.070
Adenoma dysplasia						
Low grade	16,504	82	152,448	54 (43–67)	0.8 (0.6–1.0)	
High grade	1927	23	16,695	138 (92–207)	2.9 (1.6–5.2)	<0.001
Adenoma location						
Distal only	10,887	63	102,967	61 (48–78)	1.0 (0.7–1.3)	
Any proximal	7544	42	66,176	63 (47–86)	1.0 (0.6–1.7)	0.821

^a^At 15 years of follow-up.

^b^*p*-values calculated with the log-rank test comparing cumulative incidence for adenoma characteristic subgroups.

**Fig. 3:**
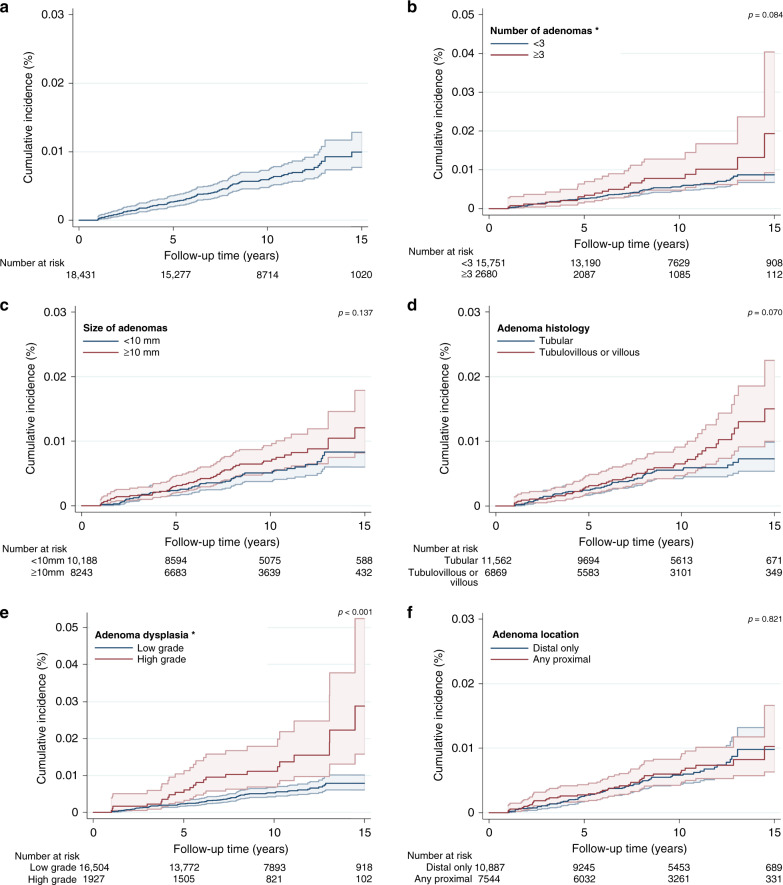
Cumulative incidence curves (%) for distal colorectal cancer within 15 years of follow-up. The graphs show Kaplan-Meier survival curves for distal colorectal cancer incidence: **a** overall; **b** by the number of adenomas; **c** by adenoma size (mm); **d** by adenoma histology; **e** by adenoma dysplasia; **f** by adenoma location. A different scale has been used in the graph for adenoma number and adenoma dysplasia and is highlighted by an asterisk. The p-values presented are for the log-rank test to compare curves for subgroups of each adenoma characteristic. The 95% CIs for each curve are shown as shaded bands.

The incidence rate of distal CRC was 62 (95% CI: 51–75) per 100,000 person-years (Table 2) and overall cumulative incidence at 15 years was 1.0% (95% CI: 0.8–1.3%) (Table 2, Fig. 3). Cumulative incidence was significantly higher for patients with adenomas with high-grade compared to low-grade dysplasia (*p* < 0.001), but no differences were observed by subgroup of adenoma number, size, histology nor location (Table 2, Fig. 3).

In crude analyses, ≥3 adenomas compared to < 3 adenomas, tubulovillous or villous histology compared to tubular, and proximal adenomas compared to only distal adenomas were associated with a higher risk of proximal colon cancer; these associations remained after adjustment for potential confounders (Table 3). When also mutually adjusting for adenoma characteristics, ≥3 adenomas compared to < 3 (adjusted HR [aHR] 2.10, 95% CI: 1.42–3.09, *p* < 0.001) and adenomas with tubulovillous or villous histology compared to tubular (aHR 1.61, 95% CI: 1.10–2.35, *p* = 0.015) were independently associated with an increased risk of incident proximal colon cancer (Table 3). Despite not being associated with distal CRC in crude or adjusted models, there was no evidence of heterogeneity in the effect of adenoma number (p-heterogeneity = 0.205) or histology (p-heterogeneity = 0.260) on CRC by subsite.

**Table 3:** Association between adenoma characteristics and proximal colon and distal colorectal cancer.

Baseline characteristics	Patients	Cases	Crude HR (95% CI)	*p*-value^a^	*p*-value het^b^	Adjusted HR (95% CI)^c^	*p*-value^a^	*p*-value het^b^	Adjusted HR (95% CI)^d^	*p-*value^a^	*p*-value het^b^
*Proximal colon cancer incidence*											
All patients	18,431	152	–			–			–		
Number of adenomas											
<3	15,751	107	1.00			1.00			1.00		
≥3	2680	45	2.56 (1.80–3.63)	<0.001	0.052	2.77 (1.94–3.94)	<0.001	0.050	2.10 (1.42–3.09)	<0.001	0.205
Adenoma size (mm)											
<10	10,188	76	1.00			1.00			1.00		
≥10	8243	76	1.26 (0.91–1.73)	0.161	0.986	1.35 (0.97–1.86)	0.072	0.992	1.04 (0.68–1.57)	0.862	0.991
Adenoma histology											
Tubular	11,562	77	1.00			1.00			1.00		
Tubulovillous or villous	6869	75	1.67 (1.21–2.30)	0.002	0.453	1.66 (1.20–2.28)	0.002	0.450	1.61 (1.10–2.35)	0.015	0.260
Adenoma dysplasia											
Low grade	16,504	133	1.00			1.00			1.00		
High grade	1927	19	1.26 (0.78–2.04)	0.342	0.051	1.29 (0.80–2.08)	0.291	0.052	1.05 (0.64–1.73)	0.833	0.023
Adenoma location											
Distal only	10,887	67	1.00			1.00			1.00		
Any proximal	7544	85	1.99 (1.44–2.74)	<0.001	0.012	2.02 (1.46–2.81)	<0.001	0.011	1.70 (1.20–2.42)	0.003	0.055
*Distal colorectal cancer incidence*											
All patients	18,431	105	–			–			–		
Number of adenomas											
<3	15,751	85	1.00			1.00			1.00		
≥3	2680	20	1.41 (0.86–2.30)	0.172		1.51 (0.92–2.49)	0.104		1.39 (0.83–2.32)	0.206	
Adenoma size (mm)											
<10	10,188	52	1.00			1.00			1.00		
≥10	8243	53	1.26 (0.86–1.85)	0.235		1.35 (0.92–1.99)	0.129		1.03 (0.62–1.73)	0.900	
Adenoma histology											
Tubular	11,562	58	1.00			1.00			1.00		
Tubulovillous or villous	6869	47	1.38 (0.94–2.03)	0.105		1.37 (0.92–2.02)	0.118		1.12 (0.68–1.84)	0.656	
Adenoma dysplasia											
Low grade	16,504	82	1.00			1.00			1.00		
High grade	1927	23	2.45 (1.54–3.90)	<0.001		2.50 (1.55–4.03)	<0.001		2.42 (1.44–4.04)	0.001	
Adenoma location											
Distal only	10,887	63	1.00			1.00			1.00		
Any proximal	7544	42	1.04 (0.70–1.53)	0.851		1.05 (0.71–1.57)	0.805		1.00 (0.66–1.52)	0.994	

*HR* hazard ratio.

^a^*p*-values calculated with the Wald test.

^b^*p*-value for heterogeneity in the effect between proximal colon and distal colorectal cancer.

^c^Adjusted for sex, age, presence of hyperplastic polyps, year of examination, bowel preparation quality, length of baseline visit and number of surveillance visits as a time-varying covariate.

^d^Mutually adjusted for each adenoma characteristic, sex, age, presence of hyperplastic polyps, year of examination, bowel preparation quality, length of baseline visit and number of surveillance visits as a time-varying covariate.

Having any proximal adenoma compared to only distal adenomas was positively and independently associated with proximal colon cancer (aHR 1.70, 95% CI: 1.20–2.42, *p* = 0.003) but was not associated with distal CRC with borderline evidence of a difference in the effect by subsite (p-heterogeneity = 0.055). Conversely, having had adenomas displaying high-grade dysplasia at baseline, compared to those with only adenomas displaying low-grade dysplasia, was independently associated with an increased risk of distal CRC incidence (aHR 2.42, 95% CI: 1.44–4.04, *p* = 0.001) but was not associated with proximal colon cancer risk (p-heterogeneity = 0.023) (Table 3).

The median time to diagnosis among patients with proximal adenomas and at least one other adenoma characteristic which was independently associated with proximal colon cancer was 5.2 (IQR 3.0–8.1) compared to 6.9 (IQR 3.9–10.2) years for those with proximal adenomas as the only characteristic detected (Appendix table 5). For patients with ≥3 adenomas, the median time to diagnosis was 5.4 (IQR 3.0–9.3) (including patients with ≥1 other adenoma characteristic) compared to 6.2 (IQR 4.6–7.9) years for patients with ≥3 adenomas alone, although there were only two cases in this group. For patients with tubulovillous or villous adenomas this was 4.8 (IQR 2.6–9.4) (including patients with ≥1 other adenoma characteristic) compared to 7.1 (IQR 3.9–9.7) years for patients with tubulovillous or villous adenomas alone (Appendix Table 5).

In sensitivity analyses, results were similar after excluding patients diagnosed with both proximal colon cancer and distal CRC (Appendix table 6). Additionally, reclassifying proximal colon and distal colorectal subsite definitions did not materially alter findings with the exception that adenoma number was now associated with distal CRC (Appendix Table 7). In both analyses, there was also stronger evidence of heterogeneity in the association between adenoma location and CRC by subsite. 

In secondary multivariable analyses adjusted for potential confounders previously identified, the presence of high-risk findings, as defined by the 2020 UK surveillance guidelines, was positively associated with proximal colon cancer (aHR 1.51, 95% CI: 1.05–2.16). However, upon the inclusion of adenoma number, histology or location to the multivariable model, this association was no longer statistically significant. Adenoma number (*p* < 0.001), histology (*p* = 0.032) and location (*p* = 0.001) were also all observed to be independently associated with proximal colon cancer in these models (data not shown).

## Discussion

To our knowledge, this is the largest cohort study to investigate the association between adenoma characteristics and the long-term incidence of proximal colon cancer compared to distal CRC among patients with adenomas at baseline colonoscopy. It revealed that, post-polypectomy, multiple (≥3) adenomas, tubulovillous or villous adenomas and proximal adenomas were independently associated with future proximal colon cancer but not with distal CRC, with borderline evidence of heterogeneity between CRC subsites for adenoma location. Adenoma dysplasia was associated with distal CRC whereas there was no association with proximal colon cancer and there was evidence of subsite heterogeneity.

The finding that proximal adenoma location was strongly associated with increased long-term proximal colon cancer incidence provides great insight into the natural history of proximal colon cancer. This lends weight to the idea that some proximal cancers arise via the adenoma-carcinoma pathway, and that the development of CRC via this pathway in the proximal colon may also be differential compared to that in the distal colorectum. This is supported by findings of a greater likelihood of proximally located metachronous adenomas after the detection of only proximal baseline adenomas compared to distal adenoma recurrence following the detection of only distal adenomas at baseline from a study based on data from three adenoma prevention trials and focused on adenoma recurrence as an outcome [3].

This current study also suggests that adenoma dysplasia may play a greater role in the development of distal CRC compared to proximal colon cancer but the small number of patients with baseline adenomas with high-grade dysplasia means that these results should be interpreted with caution. The higher risk of proximal colon cancer among patients with multiple (≥3) adenomas or tubulovillous or villous adenomas detected could also be reflective of a greater propensity for adenoma and cancer development [28] or of an increased likelihood for the development of more aggressive adenomas with faster progression rates to malignancy in these patients.

There is a greater likelihood for missed adenomas to occur in the proximal colon compared to the distal colorectum, resulting in post-polypectomy CRC [29, 30]. In order to mitigate some of the effects that poor colonoscopy quality might have on the observed associations, particularly in the proximal colon, our analysis was restricted to patients with a complete colonoscopy performed after the year 2000 when endoscopic quality criteria and methods to improve detection were introduced in the UK [31].

There are few studies investigating associations between adenoma characteristics at baseline colonoscopy and CRC, with even fewer conducting analysis of CRC by subsite [14–16]. To our knowledge, the only other study to investigate the association between polyp (including adenomas) characteristics (number, size, histology, dysplasia, location) at baseline colonoscopy and proximal colon cancer risk specifically was a case-control study conducted in Germany [2]. In line with our findings, this study found that the presence of multiple (≥3) polyps was associated with an over two-fold greater odds of proximal colon cancer. It also reported an increased risk of proximal colon cancer in patients with at least one proximal polyp at baseline compared to only distal polyps, but this was not statistically significant. Moreover, no other statistically significant associations were reported between any of the polyp characteristics under study and proximal colon cancer or distal CRC [2], although this may have been due to the smaller number of cancer cases in each subsite (proximal colon cancer [*n* = 97], distal CRC [*n* = 59]) in this previous analysis.

The present study benefited from the use of detailed data from 17 UK hospitals with wide geographic coverage, lending weight to the generalisability of the results. The use of routinely collected data from hospitals meant that the completeness and quality of endoscopy and pathology data were representative of standard hospital practice. The number of proximal colon cancer cases accrued was high due to the large cohort and long follow-up period, affording the opportunity to report on subsite-specific analyses, which is rare in many studies of this nature. The use of national cancer and vital statistics registries for outcome data on cancers, deaths and emigration resulted in a low proportion of patients who were lost to follow-up, minimising the likelihood of attrition bias. The availability of data on follow-up surveillance visits allowed for the differential surveillance contact between exposure groups to be accounted for, which is important because surveillance affects cancer outcomes.

Limitations of our study include the possibility of some measurement error or misclassification bias associated with data collected by hospitals. Data on endoscopist performance were not available for inclusion in analysis; however, as outcome data was unknown at the time of baseline colonoscopy, any bias is likely to have been non-differential leading to an attenuation of effect estimates. Data on the reason for the baseline colonoscopy referral were unavailable and therefore we were not able to disentangle any differences in the associations between adenoma characteristics in patients who were asymptomatic (i.e. attending screening) and those symptomatic. Moreover, some patients may have had a colonoscopy performed prior to our baseline, at which adenomas may have been removed thus reducing their risk as compared to patients for whom our baseline was their first colonoscopy; this may have affected our results if this was differential by exposure subgroups. Missing data for indicators of colonoscopy quality may also have biased results; however, the proportion of missingness was similar among patients with and without the main outcome. The exclusion of cancers thought to have arisen from incompletely resected adenomas at baseline could have resulted in an underestimation of the true effect of some adenoma characteristics on proximal colon cancer incidence in clinical practice.

Surveillance following baseline colonoscopy may have affected the risk of subsequent cancer in patients attending compared to those not attending. In this population of patients with baseline adenomas, among those who had follow-up surveillance colonoscopies, the majority attended within 5 years of their baseline visit, which was in line with recommendations at the time for patients with low- (5-year surveillance), intermediate- (3-year surveillance) or high-risk (1-year surveillance) adenomas at baseline [25]. Patients with adenomas which were numerous, large, tubulovillous or villous, or with high-grade dysplasia were more likely to have had a follow-up surveillance visit; this may have affected estimates in two divergent ways. These patients may have had other adenomas detected and removed during follow-up, reducing their risk of future cancer and resulting in an underestimation of the association between adenoma characteristics and CRC. Alternatively, they may have been more prone to surveillance bias, where having these characteristics may have increased their likelihood of having cancer detected due to greater contact with health services, resulting in an overestimation of the association between adenoma characteristics and CRC. Adjustment for the number of surveillance visits would have accounted for some of this in analyses but there is still the possibility of residual confounding due to differential surveillance regimes between subgroups of exposures.

The contribution of serrated polyps to CRC incidence via the serrated pathway has recently been recognised [32–35], with growing evidence of an increased likelihood of serrated polyps occurring post-colonoscopy, especially in the proximal colon [36], and with an increase in risk similar to that of conventional adenomas [37]. A lack of consistent recording by endoscopists in the era of this study precluded an examination of the separate impact of serrated lesions on proximal colon cancer incidence. Therefore, a proportion of the observed cancers in the proximal colon may have resulted from the development of serrated polyps, which were not detected or reported during baseline colonoscopy. The impact of serrated polyps in the proximal colon deserves further exploration to disentangle the effect of these polyps on proximal colon cancer incidence compared to adenomas.

Villousness is considered a criterion for colonoscopy surveillance in US guidance based on ‘moderate’ evidence of an association with CRC [15]. However, in the UK, due to inconsistencies in the classification of adenoma histology between pathologists, the strength of the evidence was not considered sufficient to support the increased resources which would be required for surveillance [14].

UK and US post-polypectomy surveillance guidelines [14, 15] do not consider adenoma location as an independent criterion for referral for surveillance, with patients either receiving no surveillance (UK) [14] or surveillance 7–10 years (US) [15] after baseline if no other high-risk adenoma characteristics (based on number, size, histology or dysplasia) are detected at baseline. This decision was based on a lack of consistent evidence to support differential management of patients with proximal adenomas [14, 15] and underpinned the recommendation for more research to determine whether these patients should be referred for surveillance [15]. In this UK cohort, the median time to diagnosis in patients with only proximal adenomas was 7 years but this was based on small numbers and should be interpreted with caution. Secondary analyses examining patients at high-risk (according to UK surveillance criteria) showed that adenoma number, histology and location were still independently associated with proximal colon cancer even after accounting for high-risk findings. However, as noted earlier, at the time of this study there was a lack of consistent reporting of serrated polyps, which are included as criteria for the classification of high-risk findings and which are thought to be important in proximal colon cancer development specifically.

This present study, in a cohort of patients followed up after a colonoscopy with polypectomy, has found that proximal location of adenomas, independent of number, size, histology or dysplasia (factors on which current guidelines are based), plays an important role in subsequent proximal colon cancer incidence. It supports previous analyses in this data reporting an increased risk of all-site CRC among patients with proximal polyps at baseline and adds to the growing body of research examining heterogeneity in risk factors for CRC by subsite. In addition, it provides much-needed insight into the specific adenoma risk factors for proximal colon cancer, a malignancy for which screening and surveillance have been less effective at reducing incidence.

## Supplementary information

Supplementary tables

STROBE checklist

## Data Availability

We may be permitted to share de-identified patient data with researchers upon reasonable request, but this will require approval from third party data providers. Requests for data should be directed to the senior author of this manuscript, Professor Amanda Cross, Cancer Screening and Prevention Research Group, Imperial College London.
