# Supplementary material for: Adenoma characteristics associated with post-polypectomy proximal colon cancer incidence: a retrospective cohort study
Source: Br J Cancer. 2022 Feb 11;126(12):1744–54. doi: 10.1038/s41416-022-01719-4 (PMC9174477; doi:10.1038/s41416-022-01719-4)
Supplement: Supplementary file 1 — Supplementary tables [file 41416_2022_1719_MOESM1_ESM.docx]

**Appendix table 1: Number and proportion of cancer cases by subsite of the colorectum**

| **Subsite** | **Number of cases** | **Proportion (%)** |
| --- | --- | --- |
| Proximal colon | 152 |  |
| Caecum | 57 | 37.5 |
| Appendix | 3 | 2.0 |
| Ascending colon | 41 | 27.0 |
| Hepatic flexure of colon | 18 | 11.8 |
| Transverse colon | 24 | 15.8 |
| Splenic flexure of colon | 9 | 5.9 |
| Distal colorectal | 105 |  |
| Descending colon | 6 | 5.7 |
| Sigmoid colon | 29 | 27.6 |
| Rectosigmoid junction | 8 | 7.6 |
| Rectum | 61 | 58.1 |
| Anus | 1 | 1.0 |

**Appendix table 2: Time interval to surveillance visit by surveillance visit number**

|  |  |  | **Time interval to surveillance visit** | | | | | | | | |
| --- | --- | --- | --- | --- | --- | --- | --- | --- | --- | --- | --- |
| **Surveillance visit number** | **All patients** |  | **≤1 year** |  | **>1-3 years** |  | **>3-5 years** |  | **>5 years** |  | **Unknown** |
| 1 | 9,613 |  | 5.9 |  | 40.3 |  | 34.8 |  | 19.0 |  | 0.0 |
| 2 | 4,491 |  | 2.9 |  | 38.9 |  | 43.7 |  | 14.5 |  | 0.0 |
| 3 | 1,511 |  | 3.8 |  | 45.6 |  | 40.0 |  | 10.5 |  | 0.0 |
| 4 | 427 |  | 5.2 |  | 56.2 |  | 31.6 |  | 7.0 |  | 0.0 |
| 5 | 95 |  | 4.2 |  | 64.2 |  | 25.3 |  | 5.3 |  | 1.1 |
| 6 | 21 |  | 9.5 |  | 66.7 |  | 23.8 |  | 0.0 |  | 0.0 |
| 7 | 6 |  | 0.0 |  | 50.0 |  | 50.0 |  | 0.0 |  | 0.0 |
| 8 | 1 |  | 100.0 |  | 0.0 |  | 0.0 |  | 0.0 |  | 0.0 |
| 9 | 1 |  | 0.0 |  | 100.0 |  | 0.0 |  | 0.0 |  | 0.0 |

**Appendix table 3: Time to first surveillance by adenoma characteristics at baseline**

|  |  |  |  | **Adenoma characteristics** | | | | | | | | | | | | | |
| --- | --- | --- | --- | --- | --- | --- | --- | --- | --- | --- | --- | --- | --- | --- | --- | --- | --- |
|  |  |  |  | **Number** | |  | **Size** | |  | **Histology** | |  | **Dysplasia** | |  | **Location** | |
|  | **All patients** | |  | **<3** | **≥3** |  | **<10mm** | **≥10mm** |  | **Tubular** | **Tubulovillous/villous** |  | **Low grade** | **High grade** |  | **Distal only** | **Any proximal** |
| **All patients (N,%)** | 9,613 | 100.0 |  | 7,904  (82.2) | 1,709  (17.8) |  | 4,694  (48.8) | 4,919  (51.2) |  | 5,617  (58.4) | 3,996  (41.6) |  | 8,400  (87.4) | 1,213  (12.6) |  | 5,684  (59.1) | 3,929  (40.9) |
| **Time to first surveillance visit, %** | |  |  |  |  |  |  |  |  |  |  |  |  |  |  |  |  |
| ≤1 year | 571 | 5.9 |  | 4.2 | 13.9 |  | 3.7 | 8.1 |  | 4.5 | 7.9 |  | 5.2 | 11.0 |  | 4.2 | 8.5 |
| >1-3 years | 3,878 | 40.3 |  | 37.7 | 52.5 |  | 34.3 | 46.1 |  | 37.1 | 44.9 |  | 38.3 | 54.7 |  | 38.1 | 43.5 |
| >3-5 years | 3,341 | 34.8 |  | 36.8 | 25.1 |  | 35.2 | 34.3 |  | 35.6 | 33.6 |  | 36.0 | 26.0 |  | 37.3 | 31.0 |
| >5 years | 1,822 | 19.0 |  | 21.2 | 8.5 |  | 26.8 | 11.5 |  | 22.8 | 13.6 |  | 20.5 | 8.3 |  | 20.4 | 16.9 |
| Unknown | 1 | 0.0 |  | 0.0 | 0.0 |  | 0.0 | 0.0 |  | 0.0 | 0.0 |  | 0.0 | 0.0 |  | 0.0 | 0.0 |

**Appendix table 4: Baseline patient and examination characteristics and number of follow-up visits by colorectal cancer outcome**

|  | **All participants** | |  | **No colorectal cancer** | |  | **Proximal colon cancer** | |  | **Distal colorectal cancer** | |
| --- | --- | --- | --- | --- | --- | --- | --- | --- | --- | --- | --- |
|  | **(N = 18,431)** | |  | **(N = 18,152)** | |  | **(N = 152)** | |  | **(N = 105)** | |
|  | **n** | **%** |  | **n** | **%** |  | **n** | **%** |  | **n** | **%** |
| **Baseline characteristics** | | | | | | | | | | | |
| **Sex** |  |  |  |  |  |  |  |  |  |  |  |
| Men | 10,755 | 58.4 |  | 10,593 | 58.4 |  | 81 | 53.3 |  | 71 | 67.6 |
| Women | 7,676 | 41.6 |  | 7,559 | 41.6 |  | 71 | 46.7 |  | 34 | 32.4 |
| **Age group (years)** |  |  |  |  |  |  |  |  |  |  |  |
| <55 | 3,586 | 19.5 |  | 3,563 | 19.6 |  | 11 | 7.2 |  | 12 | 11.4 |
| 55-64 | 5,036 | 27.3 |  | 4,978 | 27.4 |  | 33 | 21.7 |  | 25 | 23.8 |
| 65-74 | 6,020 | 32.7 |  | 5,902 | 32.5 |  | 68 | 44.7 |  | 43 | 41.0 |
| ≥75 | 3,789 | 20.6 |  | 3,709 | 20.4 |  | 40 | 26.3 |  | 25 | 23.8 |
| **Year of examination** |  |  |  |  |  |  |  |  |  |  |  |
| 2001-2005 | 7,975 | 43.3 |  | 7,828 | 43.1 |  | 86 | 56.6 |  | 50 | 47.6 |
| 2006-2010 | 10,456 | 56.7 |  | 10,324 | 56.9 |  | 66 | 43.4 |  | 55 | 52.4 |
| **Bowel preparation quality** |  |  |  |  |  |  |  |  |  |  |  |
| Excellent or good | 6,490 | 35.2 |  | 6,378 | 35.1 |  | 62 | 40.8 |  | 40 | 38.1 |
| Satisfactory | 3,749 | 20.3 |  | 3,690 | 20.3 |  | 33 | 21.7 |  | 23 | 21.9 |
| Poor | 1,071 | 5.8 |  | 1,052 | 5.8 |  | 6 | 3.9 |  | 11 | 10.5 |
| Unknown | 7,121 | 38.6 |  | 7,032 | 38.7 |  | 51 | 33.6 |  | 31 | 29.5 |
| **Length of baseline visit** |  |  |  |  |  |  |  |  |  |  |  |
| 1 day | 12,199 | 66.2 |  | 12,038 | 66.3 |  | 85 | 55.9 |  | 62 | 59.0 |
| 2 days-3 months | 2,800 | 15.2 |  | 2,748 | 15.1 |  | 27 | 17.8 |  | 21 | 20.0 |
| 3-6 months | 1,804 | 9.8 |  | 1,771 | 9.8 |  | 22 | 14.5 |  | 9 | 8.6 |
| ≥6 months | 1,628 | 8.8 |  | 1,595 | 8.8 |  | 18 | 11.8 |  | 13 | 12.4 |
| **Hyperplastic polyps** |  |  |  |  |  |  |  |  |  |  |  |
| None/Only adenomas | 14,623 | 79.3 |  | 14,418 | 79.4 |  | 105 | 69.1 |  | 81 | 77.1 |
| ≥1 | 3,808 | 20.7 |  | 3,734 | 20.6 |  | 47 | 30.9 |  | 24 | 22.9 |
| **Number of adenomas** |  |  |  |  |  |  |  |  |  |  |  |
| <3 | 15,751 | 85.5 |  | 15,544 | 85.6 |  | 107 | 70.4 |  | 85 | 81.0 |
| ≥3 | 2,680 | 14.5 |  | 2,608 | 14.4 |  | 45 | 29.6 |  | 20 | 19.0 |
| **Adenoma size (mm)** |  |  |  |  |  |  |  |  |  |  |  |
| <10 | 10,188 | 55.3 |  | 10,051 | 55.4 |  | 76 | 50.0 |  | 52 | 49.5 |
| ≥10 | 8,243 | 44.7 |  | 8,101 | 44.6 |  | 76 | 50.0 |  | 53 | 50.5 |
| **Adenoma histology** |  |  |  |  |  |  |  |  |  |  |  |
| Tubular | 11,562 | 62.7 |  | 11,414 | 62.9 |  | 77 | 50.7 |  | 58 | 55.2 |
| Tubulovillous/villous | 6,869 | 37.3 |  | 6,738 | 37.1 |  | 75 | 49.3 |  | 47 | 44.8 |
| **Adenoma dysplasia** |  |  |  |  |  |  |  |  |  |  |  |
| Low grade | 16,504 | 89.5 |  | 16,273 | 89.6 |  | 133 | 87.5 |  | 82 | 78.1 |
| High grade | 1,927 | 10.5 |  | 1,879 | 10.4 |  | 19 | 12.5 |  | 23 | 21.9 |
| **Adenoma location** |  |  |  |  |  |  |  |  |  |  |  |
| Distal only | 10,887 | 59.1 |  | 10,751 | 59.2 |  | 67 | 44.1 |  | 63 | 60.0 |
| Any proximal | 7,544 | 40.9 |  | 7,401 | 40.8 |  | 85 | 55.9 |  | 42 | 40.0 |
| **Follow-up characteristics** | | | | | | | | | | | |
| **Surveillance visits** |  |  |  |  |  |  |  |  |  |  |  |
| None | 8,818 | 47.8 |  | 8,637 | 47.6 |  | 94 | 61.8 |  | 74 | 70.5 |
| 1 | 5,122 | 27.8 |  | 5,052 | 27.8 |  | 45 | 29.6 |  | 20 | 19.0 |
| 2 | 2,980 | 16.2 |  | 2,962 | 16.3 |  | 7 | 4.6 |  | 8 | 7.6 |
| ≥3 | 1,511 | 8.2 |  | 1,501 | 8.3 |  | 6 | 3.9 |  | 3 | 2.9 |

**Appendix table 5: Median time to diagnosis of proximal colon cancer for adenoma characteristics shown to be independently associated with proximal colon cancer**

| **Baseline characteristics** | **Patients** | **Proximal colon cancer** | |
| --- | --- | --- | --- |
|  |  | **Cases** | **Time to diagnosis (years)**  **Median (IQR)** |
| **All patients** | 18,431 | 152 | 5.9 (3.3-9.3) |
| **Multiple (≥3) adenomas** |  |  |  |
| All patients with multiple adenomas | 2,680 | 45 | 5.4 (3.3-8.1) |
| Patients with multiple adenomas and ≥1 other adenoma characteristic ^a^ | 2,399 | 43 | 5.4 (3.0-9.3) |
| Patients with multiple adenomas as only characteristic detected | 281 | 2 | 6.2 (4.6-7.9) |
| **Tubulovillous or villous adenomas** |  |  |  |
| All patients with tubulovillous or villous adenomas | 6,869 | 75 | 5.5 (3.0-9.7) |
| Patients with tubulovillous or villous adenomas and ≥1 other adenoma characteristic ^b^ | 2,792 | 42 | 4.8 (2.6-9.4) |
| Patients with tubulovillous or villous adenomas as only characteristic detected | 4,077 | 33 | 7.1 (3.9-9.7) |
| **Proximal adenomas** |  |  |  |
| All patients with proximal adenomas | 7,544 | 85 | 5.5 (3.1-9.4) |
| Patients with proximal adenomas and ≥1 other adenoma characteristic ^c^ | 3,496 | 53 | 5.2 (3.0-8.1) |
| Patients with proximal adenomas as only characteristic detected | 4,048 | 32 | 6.9 (3.9-10.2) |

^a^ Tubulovillous/villous adenomas or proximal adenomas

^b^ Multiple (≥3) adenomas or proximal adenomas

^c^ Multiple (≥3) adenomas or tubulovillous/villous adenomas

**Appendix table 6: Association between adenoma characteristics and proximal colon and distal colorectal cancer excluding patients diagnosed with cancer in both subsites**

| **Baseline characteristics** | **Participants** | **Cases** | **Crude HR**  **(95% CI)** | **p value ^a^** | **p value het ^b^** | **Adjusted HR**  **(95% CI) ^c^** | **p value ^a^** | **p value het ^b^** | **Adjusted HR (95% CI) ^d^** | **p value ^a^** | **p value**  **het ^b^** |
| --- | --- | --- | --- | --- | --- | --- | --- | --- | --- | --- | --- |
| **Proximal colon cancer incidence** | | | | | | | | | | | |
| **All patients** | 18,428 | 149 | - |  |  | - |  |  | - |  |  |
| **Number of adenomas** |  |  |  |  |  |  |  |  |  |  |  |
| <3 | 15,749 | 105 | 1.00 |  |  | 1.00 |  |  | 1.00 |  |  |
| ≥3 | 2,679 | 44 | 2.55 (1.79 - 3.63) | <0.001 | 0.054 | 2.74 (1.92 – 3.92) | <0.001 | 0.052 | 2.04 (1.39 – 2.99) | <0.001 | 0.237 |
| **Adenoma size (mm)** |  |  |  |  |  |  |  |  |  |  |  |
| <10 | 10,185 | 73 | 1.00 |  |  | 1.00 |  |  | 1.00 |  |  |
| ≥10 | 8,243 | 76 | 1.31 (0.95 - 1.81) | 0.102 | 0.888 | 1.41 (1.02 - 1.96) | 0.040 | 0.883 | 1.11 (0.73 - 1.69) | 0.619 | 0.834 |
| **Adenoma histology** |  |  |  |  |  |  |  |  |  |  |  |
| Tubular | 11,561 | 76 | 1.00 |  |  | 1.00 |  |  | 1.00 |  |  |
| Tubulovillous or villous | 6,867 | 73 | 1.65 (1.19 - 2.27) | 0.002 | 0.489 | 1.64 (1.18 - 2.26) | 0.003 | 0.486 | 1.54 (1.05 - 2.26) | 0.027 | 0.322 |
| **Adenoma dysplasia** |  |  |  |  |  |  |  |  |  |  |  |
| Low grade | 16,501 | 130 | 1.00 |  |  | 1.00 |  |  | 1.00 |  |  |
| High grade | 1,927 | 19 | 1.29 (0.80 - 2.09) | 0.298 | 0.059 | 1.32 (0.82 - 2.13) | 0.248 | 0.061 | 1.07 (0.65 - 1.76) | 0.787 | 0.025 |
| **Adenoma location** |  |  |  |  |  |  |  |  |  |  |  |
| Distal only | 10,885 | 65 | 1.00 |  |  | 1.00 |  |  | 1.00 |  |  |
| Any proximal | 7,543 | 84 | 2.03 (1.47 - 2.80) | <0.001 | 0.010 | 2.05 (1.47 - 2.86) | <0.001 | 0.010 | 1.75 (1.23 - 2.48) | 0.002 | 0.042 |
| **Distal colorectal cancer incidence** | | | | | | | | | | | |
| **All patients** | 18,428 | 105 | - |  |  | - |  |  | - |  |  |
| **Number of adenomas** |  |  |  |  |  |  |  |  |  |  |  |
| <3 | 15,749 | 85 | 1.00 |  |  | 1.00 |  |  | 1.00 |  |  |
| ≥3 | 2,679 | 20 | 1.41 (0.86 - 2.30) | 0.172 |  | 1.50 (0.92 – 2.47) | 0.107 |  | 1.39 (0.83 - 2.32) | 0.208 |  |
| **Adenoma size (mm)** |  |  |  |  |  |  |  |  |  |  |  |
| <10 | 10,185 | 52 | 1.00 |  |  | 1.00 |  |  | 1.00 |  |  |
| ≥10 | 8,243 | 53 | 1.26 (0.86 - 1.85) | 0.235 |  | 1.36 (0.92 – 2.00) | 0.124 |  | 1.04 (0.62 – 1.74) | 0.895 |  |
| **Adenoma histology** |  |  |  |  |  |  |  |  |  |  |  |
| Tubular | 11,561 | 58 | 1.00 |  |  | 1.00 |  |  | 1.00 |  |  |
| Tubulovillous or villous | 6,867 | 47 | 1.38 (0.94 - 2.03) | 0.105 |  | 1.37 (0.93 – 2.02) | 0.116 |  | 1.12 (0.68 - 1.85) | 0.649 |  |
| **Adenoma dysplasia** |  |  |  |  |  |  |  |  |  |  |  |
| Low grade | 16,501 | 82 | 1.00 |  |  | 1.00 |  |  | 1.00 |  |  |
| High grade | 1,927 | 23 | 2.46 (1.54 - 3.90) | <0.001 |  | 2.51 (1.56 – 4.04) | <0.001 |  | 2.42 (1.44 – 4.05) | 0.001 |  |
| **Adenoma location** |  |  |  |  |  |  |  |  |  |  |  |
| Distal only | 10,885 | 63 | 1.00 |  |  | 1.00 |  |  | 1.00 |  |  |
| Any proximal | 7,543 | 42 | 1.04 (0.70 - 1.53) | 0.851 |  | 1.05 (0.70 - 1.56) | 0.825 |  | 1.00 (0.66 - 1.52) | 0.989 |  |

HR=Hazard ratio

^a^ p values calculated with the Wald test

^b^ p value for heterogeneity in the effect between proximal colon and distal colorectal cancer

^c^ adjusted for sex, age, presence of hyperplastic polyps, year of examination, bowel preparation quality, length of baseline visit and number of surveillance visits as a time-varying covariate

^d^ mutually adjusted for each adenoma characteristic, sex, age, presence of hyperplastic polyps, year of examination, bowel preparation quality, length of baseline visit and number of surveillance visits as a time-varying covariate

**Appendix table 7: Association between adenoma characteristics and proximal colon cancer and distal colorectal cancer – alternative subsite definitions ***

| **Baseline characteristics** | **Participants** | **Cases** | **Crude HR**  **(95% CI)** | **p value ^a^** | **p value het ^b^** | **Adjusted HR**  **(95% CI) ^c^** | **p value ^a^** | **p value het ^b^** | **Adjusted HR (95% CI) ^d^** | **p value ^a^** | **p value**  **het ^b^** |
| --- | --- | --- | --- | --- | --- | --- | --- | --- | --- | --- | --- |
| **Proximal colon cancer incidence** | | | | | | | | | | | |
| **All patients** | 18,431 | 119 | - |  |  | - |  |  | - |  |  |
| **Number of adenomas** |  |  |  |  |  |  |  |  |  |  |  |
| <3 | 15,751 | 84 | 1.00 |  |  | 1.00 |  |  | 1.00 |  |  |
| ≥3 | 2,680 | 35 | 2.52 (1.69 - 3.75) | <0.001 | 0.160 | 2.72 (1.82 – 4.07) | <0.001 | 0.156 | 2.06 (1.32 – 3.22) | 0.001 | 0.559 |
| **Adenoma size (mm)** |  |  |  |  |  |  |  |  |  |  |  |
| <10 | 10,188 | 60 | 1.00 |  |  | 1.00 |  |  | 1.00 |  |  |
| ≥10 | 8,243 | 59 | 1.23 (0.86 - 1.76) | 0.259 | 0.868 | 1.32 (0.92 - 1.90) | 0.137 | 0.864 | 1.02 (0.64 - 1.61) | 0.936 | 0.974 |
| **Adenoma histology** |  |  |  |  |  |  |  |  |  |  |  |
| Tubular | 11,562 | 60 | 1.00 |  |  | 1.00 |  |  | 1.00 |  |  |
| Tubulovillous or villous | 6,869 | 59 | 1.68 (1.17 - 2.41) | 0.005 | 0.530 | 1.67 (1.16 - 2.39) | 0.006 | 0.529 | 1.65 (1.07 - 2.52) | 0.022 | 0.289 |
| **Adenoma dysplasia** |  |  |  |  |  |  |  |  |  |  |  |
| Low grade | 16,504 | 105 | 1.00 |  |  | 1.00 |  |  | 1.00 |  |  |
| High grade | 1,927 | 14 | 1.18 (0.67 - 2.06) | 0.568 | 0.070 | 1.20 (0.69 - 2.09) | 0.516 | 0.070 | 0.97 (0.55 - 1.72) | 0.917 | 0.039 |
| **Adenoma location** |  |  |  |  |  |  |  |  |  |  |  |
| Distal only | 13,082 | 65 | 1.00 |  |  | 1.00 |  |  | 1.00 |  |  |
| Any proximal | 5,349 | 54 | 2.22 (1.54 - 3.19) | <0.001 | 0.005 | 2.20 (1.52 – 3.18) | <0.001 | 0.005 | 1.86 (1.25 - 2.78) | 0.002 | 0.013 |
| **Distal colorectal cancer incidence** | | | | | | | | | | | |
| **All patients** | 18,431 | 138 | - |  |  | - |  |  | - |  |  |
| **Number of adenomas** |  |  |  |  |  |  |  |  |  |  |  |
| <3 | 15,751 | 108 | 1.00 |  |  | 1.00 |  |  | 1.00 |  |  |
| ≥3 | 2,680 | 30 | 1.68 (1.12 - 2.52) | 0.013 |  | 1.80 (1.20 – 2.72) | 0.005 |  | 1.72 (1.14 - 2.60) | 0.009 |  |
| **Adenoma size (mm)** |  |  |  |  |  |  |  |  |  |  |  |
| <10 | 10,188 | 68 | 1.00 |  |  | 1.00 |  |  | 1.00 |  |  |
| ≥10 | 8,243 | 70 | 1.28 (0.92 - 1.79) | 0.145 |  | 1.38 (0.98 - 1.94) | 0.067 |  | 1.03 (0.66 – 1.62) | 0.898 |  |
| **Adenoma histology** |  |  |  |  |  |  |  |  |  |  |  |
| Tubular | 11,562 | 75 | 1.00 |  |  | 1.00 |  |  | 1.00 |  |  |
| Tubulovillous or villous | 6,869 | 63 | 1.44 (1.03 - 2.01) | 0.035 |  | 1.42 (1.01 – 2.00) | 0.042 |  | 1.19 (0.77 - 1.82) | 0.434 |  |
| **Adenoma dysplasia** |  |  |  |  |  |  |  |  |  |  |  |
| Low grade | 16,504 | 110 | 1.00 |  |  | 1.00 |  |  | 1.00 |  |  |
| High grade | 1,927 | 28 | 2.24 (1.48 - 3.39) | <0.001 |  | 2.29 (1.49 – 3.51) | <0.001 |  | 2.11 (1.33 – 3.33) | 0.001 |  |
| **Adenoma location** |  |  |  |  |  |  |  |  |  |  |  |
| Distal only | 13,082 | 99 | 1.00 |  |  | 1.00 |  |  | 1.00 |  |  |
| Any proximal | 5,349 | 39 | 1.05 (0.73 - 1.53) | 0.778 |  | 1.04 (0.71 - 1.53) | 0.825 |  | 0.93 (0.63 - 1.37) | 0.706 |  |

* Proximal colon cancer included cancer occurring between the caecum to hepatic flexure (International Classification of Diseases for Oncology, third edition [ICD-O-3] codes C18.0–C18.3). Distal colorectal cancer included cancer occurring between the anus and transverse colon (ICD-O-3 codes C18.4–C18.7; C19; C20; C21)

HR=Hazard ratio

^a^ p values calculated with the Wald test

^b^ p value for heterogeneity in the effect between proximal colon and distal colorectal cancer

^c^ adjusted for sex, age, presence of hyperplastic polyps, year of examination, bowel preparation quality, length of baseline visit and number of surveillance visits as a time-varying covariate

^d^ mutually adjusted for each adenoma characteristic, sex, age, presence of hyperplastic polyps, year of examination, bowel preparation quality, length of baseline visit and number of surveillance visits as a time-varying covariate
